# Literary Notes

**Published:** 1909-03

**Authors:** 


### ITEM.

Battle & Co., 2001 Locust Street, St. Louis, Mo., have issued No. 8 of their Dislocation Chart series. Physicians desiring any back numbers can get them upon request to the publishers.

